# Illuminating Relationships Between the Pre- and Post-synapse

**DOI:** 10.3389/fncir.2020.00009

**Published:** 2020-04-02

**Authors:** Thomas M. Sanderson, John Georgiou, Graham L. Collingridge

**Affiliations:** ^1^Lunenfeld-Tanenbaum Research Institute, Toronto, ON, Canada; ^2^Tanz Centre for Research in Neurodegenerative Diseases, Department of Physiology, University of Toronto, Toronto, ON, Canada; ^3^Glutamate Research Group, School of Physiology, Pharmacology and Neuroscience, University of Bristol, Bristol, United Kingdom

**Keywords:** probability of neurotransmitter release, AMPA receptor, mGluRs, presynapse, postsynapse, styryl dyes, imaging, synaptic function

## Abstract

Excitatory synapses in the mammalian cortex are highly diverse, both in terms of their structure and function. However, relationships between synaptic features indicate they are highly coordinated entities. Imaging techniques, that enable physiology at the resolution of individual synapses to be investigated, have allowed the presynaptic activity level of the synapse to be related to postsynaptic function. This approach has revealed that neuronal activity induces the pre- and post-synapse to be functionally correlated and that subsets of synapses are more susceptible to certain forms of synaptic plasticity. As presynaptic function is often examined in isolation from postsynaptic properties, the effect it has on the post-synapse is not fully understood. However, since postsynaptic receptors at excitatory synapses respond to release of glutamate, it follows that they may be differentially regulated depending on the frequency of its release. Therefore, examining postsynaptic properties in the context of presynaptic function may be a useful way to approach a broad range of questions on synaptic physiology. In this review, we focus on how optophysiology tools have been utilized to study relationships between the pre- and the post-synapse. Multiple imaging techniques have revealed correlations in synaptic properties from the submicron to the dendritic level. Optical tools together with advanced imaging techniques are ideally suited to illuminate this area further, due to the spatial resolution and control they allow.

## Introduction

Synapses in the brain are diverse, plastic structures with distinct morphologies (Walmsley et al., 1998; Rollenhagen and Lubke, 2006). Even within one type of synapse, the excitatory cortical synapse, a large degree of structural and functional heterogeneity is observed, which is likely of relevance to the information processing that contributes to memory and cognition (Harris and Stevens, 1989; Schikorski and Stevens, 1999; Matsuzaki et al., 2004; Noguchi et al., 2005; Harvey et al., 2008; Tanaka et al., 2008; Araya et al., 2014). Many experimental techniques have been utilized to understand synaptic physiology (Glasgow et al., 2019); however, light-based imaging techniques are particularly powerful for studying this area.

Ultrastructural studies help illustrate the extent of synaptic variability. They have revealed that spine head volume in the hippocampus can vary by ∼180 fold, postsynaptic density (PSD) area by ∼70 fold, spine length by ∼10 fold, and synaptic vesicle number by ∼500 fold (Harris and Stevens, 1989). The richness in synaptic form is likely a consequence of molecular composition. For example, overexpression of postsynaptic proteins such as PSD-95 (El-Husseini et al., 2000), Shank (Sala et al., 2001), and GluA2 (Passafaro et al., 2003) can drive the morphological enlargement of spines. Conversely, the specific morphologies may act to influence the molecular composition. For example, mushroom spines with smaller heads and long spine necks slow the diffusion of AMPA receptors (AMPARs) (Ashby et al., 2006), which may make the AMPAR complement in the spine head more stable.

The functionality of synapses is related to their structure and molecular composition. For example, one highly reproduced finding that mirrors the presynaptic structural diversity of synapses is that the probability of neurotransmitter release [P(r)] at central synapses is highly variable. The diversity of P(r) has been measured using a variety of methods including the progressive block by the use-dependent NMDA receptor (NMDAR) antagonist MK-801 (Hessler et al., 1993; Rosenmund et al., 1993), the activity-dependent uptake of styryl dye (Murthy et al., 1997; Sanderson et al., 2018), high affinity calcium indicators like Oregon Green BAPTA-1 (Emptage et al., 1999, 2003; Ward et al., 2006; Enoki et al., 2009; Padamsey et al., 2017, 2019) or the glutamate sensor SF-iGluSnFR (Jensen et al., 2019; Soares et al., 2019). P(r) correlates with structural features of synapses such as the active zone area (Schikorski and Stevens, 1997; Holderith et al., 2012) and also with the readily releasable pool size (Dobrunz and Stevens, 1997) which is thought to consist of those vesicles docked at the active zone (Schikorski and Stevens, 1997; Murthy et al., 2001). These findings suggest P(r) is powerfully influenced by presynaptic structural attributes.

When the post-synapse is studied, a similar correspondence between structure and function is observed. A precise relationship exists between the molecular complement of spines and their geometry. For example, the PSD length and basal AMPAR expression are positively correlated, with functional AMPARs expressed at a similar density across different spines (Takumi et al., 1999; Tanaka et al., 2005). In addition, probing synaptic function by focally uncaging caged glutamate at synapses using two-photon stimulation (Mitchell et al., 2019), has revealed that expression of glutamate receptors is correlated with spine volume (Noguchi et al., 2005) and that long-term potentiation (LTP)-associated changes in volume correlate with changes in conductance (Matsuzaki et al., 2004).

Many factors influence the relationship between postsynaptic structure and function. For example when specific proteins such as PSD-95 are knocked out, there is a resultant increase in silent synapses on mature spines (Beique et al., 2006). Additional contributions, such as astrocytes, play important roles in synapse development and function; however, a more detailed description of how they influence the pre- and post-synapse is beyond the scope of this review. The role of astrocytes in synapse physiology is described in several recent reviews (Allen and Eroglu, 2017; Rose et al., 2017; Dallerac et al., 2018). Here, we summarize some of the findings that suggest both pre- and post-synaptic activity is highly coordinated and discuss functional imaging studies that suggest multiple mechanisms are involved in ensuring the pre- and post-synaptic compartments are functionally aligned.

## Postsynaptic Manipulations Influence the Presynapse

**FIGURE 1:**
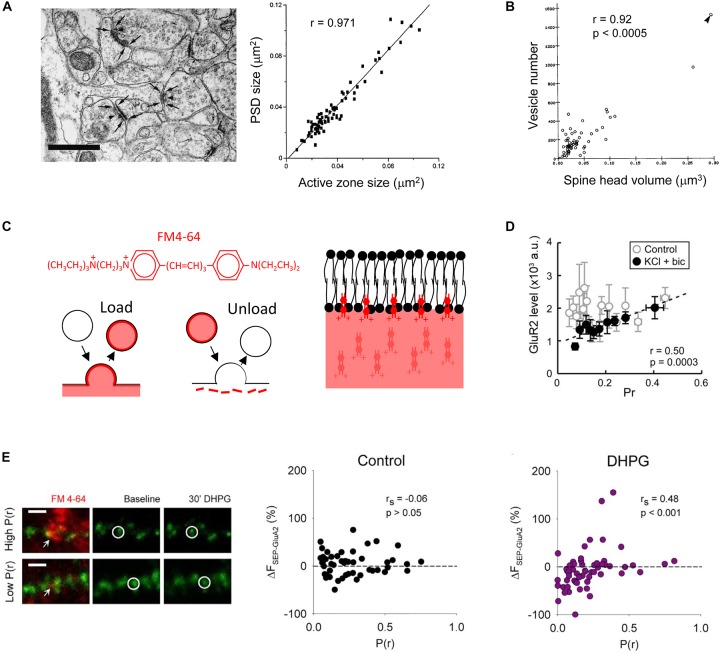
Multiple pre- and post-synaptic features are correlated. Analysis of electron micrographs indicates that **(A)** the postsynaptic density (PSD) size correlates with the presynaptic active zone size, scale bar in image on left is 0.5 μm [Schikorski and Stevens, 1997, copyright (1997) Society for Neuroscience]; and **(B)** the postsynaptic spine head volume correlates with the number of vesicles in the presynaptic varicosity [Harris and Stevens, 1989, copyright (1989) Society for Neuroscience]. **(C)** FM4-64 is a red styryl dye that allows simultaneous imaging with green fluorescent proteins. It is an amphipathic molecule that is taken into lipid membranes due to a lipophilic portion but does not fully cross due to a polar head. It can be loaded and unloaded in an activity-dependent way. Scheme based on that in Betz et al., 1992 [copyright (1992) Society for Neuroscience]. **(D)** In dissociated cultured neurons, no functional relationship is observed in control conditions when P(r) is measured using loading of FM dye and postsynaptic strength by immunofluorescence imaging against GluA2. However, if activity is elevated by application of potassium chloride and bicuculline, a correlation is observed due to the down regulation of surface GluA2 at low P(r) synapses [Tokuoka and Goda, 2008, copyright (2008) National Academy of Sciences]. **(E)** Application of the group I mGluR agonist DHPG results in downregulation of AMPARs at low P(r) synapses. P(r) estimated by FM4-64 loading (red) and postsynaptic strength indicated by SEP-GluA2 fluorescence (green). Example of FM4-64 loading and SEP-GluA2 fluorescence in images on left, scale bar represents 2 μm. Arrows indicate FM4-64 puncta used to estimate P(r). Circles indicate a SEP-GluA2 punctum at the synapse opposed to the FM4-64 labeling. Reproduced from Sanderson et al. (2018) under CC BY-NC-ND license.

Ultrastructural studies suggest that numerous features of the pre- and post-synapse are correlated. These include relationships between the PSD size and the active zone size, and between the postsynaptic spine head volume and the number of vesicles in the presynaptic varicosity (Figures 1A,B) (Harris and Stevens, 1989; Schikorski and Stevens, 1997, 1999). These interrelations in structural composition indicate there are mechanisms to ensure that as synapses are modified, for example, over development or due to synaptic plasticity, the pre- and post-synapse remain proportional to one another. In addition to driving an increase in spine size as mentioned above, overexpressing postsynaptic proteins results in enhanced miniature excitatory postsynaptic current (mEPSC) frequency, often interpreted as reflecting an increase in P(r), as well as a range of other measures of presynaptic function (El-Husseini et al., 2000; Sala et al., 2001). The presynaptic effect is indicative of a functional increase in synaptic activity that may be in proportion to the increase in postsynaptic spine size. Due to the similar density of functional AMPAR expression (Takumi et al., 1999; Tanaka et al., 2005), this would also be proportional to postsynaptic function.

Conversely, knocking down AMPARs reduces presynaptic functionality, suggested by reduced mEPSC frequency and reduced uptake of antibody directed against the vesicular protein synaptotagmin. When other measures of presynaptic activity were examined, by measuring paired pulse facilitation and MK-801 block, there was no difference detected. This led the authors to conclude that a subset of synapses had become inactive, leaving the remaining synapses with unaltered P(r) (Tracy et al., 2011). Two types of release have been identified, one responsible for mEPSCs, which does not require presynaptic action potentials, the other involved in evoked release, which does (Kavalali, 2015). Presynaptic NMDARs regulate these two forms of release differentially, for example, they may act to regulate mEPSC release via c-Jun N terminal Kinase, whereas they may regulate evoked release via Rab3-interacting molecule (RIM) 1 (Abrahamsson et al., 2017). A possible alternative interpretation of the constellation of presynaptic changes that accompany postsynaptic AMPAR knockdown (Tracy et al., 2011) is that they indicate a deficit in neurotransmitter release that does not require presynaptic action potentials.

## Pre- and Post-Synaptic Proteins Are Aligned at the Submicron Level

The ability to image synaptic proteins using super resolution imaging has resulted in the discovery of subsynaptic domains. These are ∼70–80 nm domains enriched with PSD-95, Homer, and AMPARs at the post-synapse of excitatory synapses (Fukata et al., 2013; MacGillavry et al., 2013; Nair et al., 2013; Tang et al., 2016; Hruska et al., 2018). These domains are of uniform size and are regulated by palmitoylation and by interactions with stargazin (Fukata et al., 2013; Nair et al., 2013).

Examining synapses using 3D stochastic optical reconstruction microscopy (3D-STORM) (Huang et al., 2008) has revealed that the subsynaptic domains at the post-synapse are related to the presynapse in trans-synaptic nanocolumns. These consist of enriched areas of PSD-95 and AMPARs that are located directly opposite the presynaptic release machinery characterized as containing RIM (Tang et al., 2016). Presynaptic vesicle fusion was detected preferentially at areas of RIM enrichment, suggesting that this nanoscale architecture is relevant for synaptic function. That the presynaptic release machinery relates so precisely to the postsynaptic receptors that detect release is predicted to affect the efficiency of synaptic transmission (Nair et al., 2013). For example, this organization is estimated to enhance synaptic strength by 20 % compared to if pre- and post-synaptic proteins were organized uniformly (Tang et al., 2016).

Stimulated emission depletion (STED) microscopy (Willig et al., 2006) has revealed that since trans-synaptic nanocolumns are of remarkably similar size, this means that large synapses that contain more synaptic proteins do not have larger trans-synaptic nanocolumns, but have a greater number of similarly sized domains. When structural plasticity is induced, the modular addition of new trans-synaptic nanocolumns is particularly apparent at later time points (>2 h after induction). The pre- and post-synaptic elements of trans-synaptic nanocolumns remain aligned even though they are mobile when undergoing structural plasticity (Hruska et al., 2018).

## Measurement of P(r) Using FM Dyes

In order to study pre-synapse function, imaging techniques have proved invaluable. Styryl dyes such as FM1-43 or FM4-64 have proved useful as they offer the benefits of being amenable to measuring P(r) of evoked neurotransmission directly, and can be used in semi-intact (e.g., slice) preparations. FM dyes were used originally to study vesicle recycling at the frog neuromuscular junction (Betz and Bewick, 1992; Betz et al., 1992) and the hippocampus (Ryan et al., 1993), enabling multiple presynaptic boutons to be studied simultaneously in response to direct electrical stimulation.

These amphipathic dyes are incorporated into biological lipid membranes due to their short hydrophobic tail, but do not pass all the way through the membrane due to the highly charged hydrophilic group at the opposite end of the molecule (Figure 1C) (Betz et al., 1992). When applied to biological preparations these dyes therefore stain all external membranes. Following release of neurotransmitter through fusion of a vesicle with the presynaptic membrane, the vesicular membrane and associated proteins are recycled via clathrin-dependent endocytosis (Brodin et al., 2000; Watanabe et al., 2013). If external membranes have been stained with FM dye, this recycling process results in newly recycled vesicles that are also stained with dye. Dye in external membranes can then be washed out, leaving only those internalized vesicles that can be visualized as puncta by confocal or multiphoton microscopy and when photo converted can be visualized in synaptic vesicles by electron microscopy (Harata et al., 2001; Branco et al., 2008). The development of agents that reduce background FM staining (Kay et al., 1999; Pyle et al., 1999) have enabled the use of this technique in brain slice preparations (Johnstone and Raymond, 2013; Sanderson et al., 2018) as well as facilitating their use in dissociated culture (Tokuoka and Goda, 2008).

FM dye uptake has been used in two ways to assess presynaptic activity. The first involves loading FM dye into presynaptic vesicles using a low number of electrical stimulations, a protocol that results in individual peaks in the fluorescence intensity frequency histogram that likely represent the fluorescence from individual vesicles (Murthy et al., 1997). With reference to the number of stimulations used to load the presynaptic boutons with dye, and the fluorescence value ascribed to a single vesicle, this staining procedure allows the P(r) to be estimated (Murthy et al., 1997; Tokuoka and Goda, 2008; Sanderson et al., 2018). When loaded in a hippocampal slice using a stimulating electrode the dye reveals very sparse labeling. If care is taken to ensure that the stimulation strength is similar to that used in slice electrophysiology experiments, the labeled synapses can be related to those studied using electrophysiology (Sanderson et al., 2018). In addition, FM dyes can be loaded into dissociated cultured neurons using a high concentration of potassium. This depolarizes the cells causing release followed by loading of the entire recycling pool of vesicles. Since the size of the recycling pool is correlated with P(r) (Rosenmund and Stevens, 1996; Dobrunz and Stevens, 1997; Murthy et al., 1997), this method has also been used to assess presynaptic activity levels (Kay et al., 2011).

## The Development of Functionally Correlated Pre- and Post-Synaptic Compartments

A functional correlation between pre- and post-synaptic activity has been observed under a variety of conditions. An initial connection was observed by relating the intensity of GluA1 immunofluorescence staining in dissociated culture to the uptake of antibody directed against the vesicular protein synaptotagmin (Thiagarajan et al., 2005). However, using FM dyes it was found that this functional correlation is only seen if the neuronal network exhibits sufficient activity. In dissociated cultured neurons, using loading of FM1-43 to measure P(r), and an antibody against the AMPAR subunit GluA2 as a measure of postsynaptic strength, a functional correlation emerged when neuronal activity was elevated pharmacologically (Figure 1D) (Tokuoka and Goda, 2008). Furthermore, where postsynaptic function has been assessed using focal uncaging of caged glutamate, a correlation between P(r) emerged over development, in a manner that depended on neuronal activity (Kay et al., 2011). These observations have revealed that an activity-dependent correlation between pre- and post-synaptic function emerges during development. This leads to the question as to the underlying mechanisms?

## Sep-Tagged Receptors Reveal AMPAR Trafficking Is Influenced by P(r)

Live cell imaging aimed at understanding the AMPAR trafficking that contributes to the expression of hippocampal synaptic plasticity has helped shed light on the activity-dependent mechanisms that may underlie the correspondence between pre- and post-synaptic properties. One imaging method involves using super ecliptic pHluorin (SEP), a pH-sensitive variant of Green Fluorescent Protein that yields greater fluorescence at neutral pH compared to acidic pH. SEP expression allows the preferential imaging of GluA2 at the cell surface where pH is ∼7, rather than in the endocytic pathway where pH is ∼5. Versions of this fluorophore were initially developed, and have been used extensively, to study presynaptic secretion (Miesenbock et al., 1998; Sankaranarayanan et al., 2000; Voglmaier et al., 2006; Lindskog et al., 2010; Henry et al., 2012). This methodology was then adopted for the study of postsynaptic receptor trafficking, where AMPARs were shown to rapidly internalize in response to activation of NMDARs (Ashby et al., 2004). This approach has since been used extensively to study various aspects of AMPAR receptor trafficking (Ashby et al., 2004, 2006; Kopec et al., 2006, 2007; Lin and Huganir, 2007; Yudowski et al., 2007; Heine et al., 2008; Lin et al., 2009; Araki et al., 2010, 2015; Patterson et al., 2010; Thorsen et al., 2010; Makino and Malinow, 2011; Sanderson et al., 2011, 2018; Zhang et al., 2011, 2015).

We have recently combined the use of SEP-GluA2 and FM4-64 to investigate the relationship between postsynaptic AMPAR trafficking and P(r) at individual hippocampal synapses (Sanderson et al., 2018). In particular, we studied a form of synaptic plasticity that is induced by a brief application of the group 1 mGluR agonist dihydroxyphenylglycine (DHPG), termed DHPG-long term depression (DHPG-LTD) (Palmer et al., 1997). The dual probes validated the notion that AMPAR trafficking contributes to the expression of DHPG-LTD (Snyder et al., 2001; Moult et al., 2006; Casimiro et al., 2011) and may occur only at a subset of synapses (Xiao et al., 2001; Sanderson et al., 2011). More interestingly, the optical approach also revealed that the DHPG-induced SEP-GluA2 trafficking is correlated with P(r), such that reductions in AMPARs occur predominantly at low P(r) synapses (Figure 1E).

Where manipulations have been performed that modulate P(r), for example, changing the calcium to magnesium ratio, the magnitude of DHPG-LTD and the extent of AMPAR trafficking is altered in a way that is consistent with this mechanism. For example, increasing the calcium to magnesium ratio results in higher P(r) and DHPG-LTD and SEP-GluA2 trafficking are both reduced; if the ratio is decreased to lower P(r) then LTD and GluA2 trafficking are both increased (Oliet et al., 1997; Watabe et al., 2002; Sanderson et al., 2018).

## Mechanisms of P(r)-Dependent mGluR Activation

**FIGURE 2:**
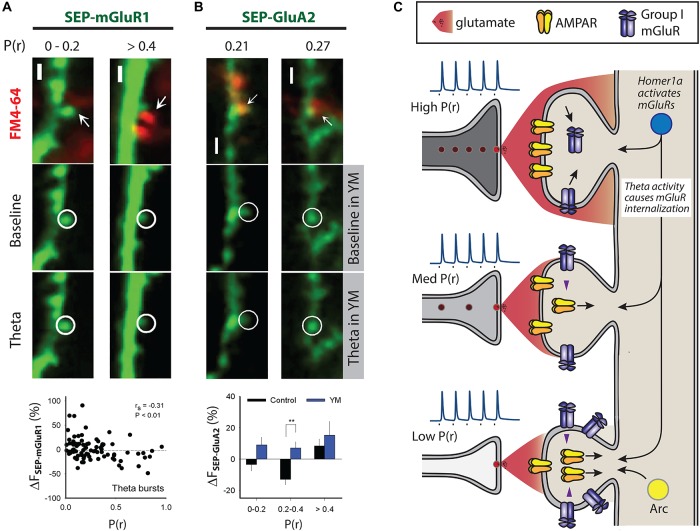
Mechanisms that may contribute to differential AMPAR trafficking. Multiphoton images in **(A)** and **(B)** show examples of SEP fluorescence (green) and FM4-64 staining (red) to estimate P(r), scale 2 μm. **(A)** Theta burst activity induces trafficking of SEP-mGluR1 in organotypic slices such that decreases are seen at high P(r) synapses. P(r) estimated by loading of FM4-64. **(B)** Theta burst activity engages mGluR1 at intermediate P(r) synapses, defined as 0.2 < P(r) < 0.4, to induce SEP-GluA2 trafficking (lower left images). P(r) estimated by loading of FM4-64, YM indicates the mGluR1 specific antagonist YM 298198 at 2 μm (lower right images). ** indicates *p* < 0.01. **(A)** and **(B)** reproduced from Sanderson et al. (2018) under CC BY-NC-ND license. **(C)** Scheme compares physiological responses to presynaptic activity at three synapses that are of low, medium and high P(r). Inset spikes are action potentials generated upon nerve stimulation (square) with relative amount of glutamate neurotransmitter released from vesicles. Pre- and post-synaptic structural features are correlated, as are functional measures, such as AMPAR expression and P(r). High P(r) synapses express fewer mGluR group I receptors due to the action of theta burst activity. Theta burst activity is also able to induce AMPAR trafficking at a subset of medium P(r) synapses (Sanderson et al., 2018). Arc is expressed at less active synapses where its expression correlates with AMPAR removal, and thus could contribute to a mechanism that would ensure lower levels of AMPARs at these synapses (Okuno et al., 2012). Homer1a is able to induce AMPAR trafficking by triggering group I mGluRs in an agonist-independent way (Hu et al., 2010). Homer1a may therefore be an effective trigger for group I mGluRs at less active synapses.

Why does DHPG-induced AMPAR trafficking occur predominantly at low P(r) synapses? DHPG-LTD can be triggered by either mGluR1 or mGluR5 (Gladding et al., 2009b; Sanderson et al., 2016). The trigger for DHPG-LTD in organotypic slices is mGluR1 (Nadif Kasri et al., 2011; Sanderson et al., 2018) and using a similar imaging approach mGluR1 was tagged with SEP in order to compare its expression and trafficking with P(r). The approach revealed that specifically at *high* P(r) synapses theta burst stimulation (TBS) induces downregulation of mGluR1 resulting in lower basal mGluR1 levels (Figure 2A) (Sanderson et al., 2018). Therefore, DHPG-induced AMPAR trafficking occurs predominantly at *low* P(r) synapses because this is where the trigger, mGluR1, is expressed most highly. As theta burst activity develops over the course of development (Charlesworth et al., 2015; Kim et al., 2016), this mechanism may contribute to the emergence of a correlation between P(r) and postsynaptic function at later developmental stages (Kay et al., 2011).

Inhibition of excitatory amino acid transporters (EAATs) was found to enhance the trafficking of SEP-mGluR1 suggesting that the selective effect of theta bursts at high P(r) synapses is likely because the greater frequency of glutamate release at these synapses results in more spillover of glutamate to peri-synaptic areas where mGluRs are expressed (Sanderson et al., 2018). Consistent with this hypothesis, STED imaging has revealed that LTP causes the withdrawal of astroglial processes from synapses, and in so doing facilitates the spillover of glutamate (Henneberger et al., 2018). Consequently, released glutamate would gain access to peri-synaptically expressed receptors such as mGluR1 and so may be necessary for the theta burst-induced mGluR1 trafficking detailed above. Whether this modulation of astroglia occurs more readily at high P(r) synapses remains to be investigated.

## Possible Functional Consequences of P(r) Influenced Ampar Trafficking

What are the possible functional consequences of a relationship between P(r) and AMPAR trafficking? Here we put forward a few suggestions.

### Theta-Burst Stimulation (TBS)

We found that TBS induces SEP-GluA2 trafficking in synapses of intermediate P(r) via a mechanism that requires mGluR1 activation (Figure 2B) (Sanderson et al., 2018). That theta bursts have this effect may indicate that mGluR-induced AMPAR trafficking can sculpt neuronal networks in an input specific way. The recruitment of this mechanism only at synapses of intermediate P(r) is likely because a balance exists between mGluR1 expression levels and sufficient release of glutamate to activate them. High P(r) synapses may be protected from the effects of mGluR1 activation due to their downregulation, and mGluRs at low P(r) synapses may not be appropriately activated due to insufficient release of glutamate. According to this hypothesis, intermediate P(r) synapses are in a “Goldilocks zone” in which the glutamate released by theta burst stimulation is sufficient to activate mGluRs to induce AMPAR trafficking, but not enough to cause the internalization of mGluRs themselves.

### Synaptic Down-Scaling

An additional potential mechanism involves mGluR activation via non glutamatergic signaling and the immediate early gene (IEG) Homer 1a (Figure 2C) (Brakeman et al., 1997; Ango et al., 2001). An increase in excitability as a result of *in vitro* application of a GABA_A_ antagonist such as bicuculline results in decreased synaptic transmission due to the cell wide downregulation of surface AMPARs, a form of homeostatic plasticity termed synaptic scaling-down (Turrigiano, 2008). The expression of Homer 1a is also driven by increases in neuronal excitability, and when expressed it activates group I mGluRs in an agonist-independent way by disrupting crosslinking of constitutively expressed versions of Homer (Brakeman et al., 1997; Ango et al., 2001; Hu et al., 2010). Homer 1a-mGluR signaling is the trigger for the AMPAR trafficking in synaptic scaling-down and overexpression of Homer 1a is able to drive AMPAR trafficking in an mGluR1/5-dependent way (Figure 2C) (Hu et al., 2010). As mGluR1 is enriched at low P(r) synapses (Sanderson et al., 2018), it is possible that AMPAR trafficking induced by Homer1a-mGluR1 signaling would be more likely to occur at low P(r) synapses. Therefore, synaptic scaling-down may also act to ensure functional registration between the pre- and post-synapse via this mechanism. Where it was found that elevated neuronal activity is needed for a correlation in pre- and post-synaptic function to emerge, a very similar protocol to that which induces synaptic scaling-down was used and the alterations in AMPAR expression were exclusively at low P(r) synapses (Figure 1D) (Tokuoka and Goda, 2008). Putting these results together presents a plausible case for mGluR-triggered AMPAR trafficking that is engaged by elevated neuronal activity and that acts at low P(r) synapses to ensure a functional correlation between the pre- and post-synapse.

As direct pharmacological activation of group I mGluRs using DHPG also results in AMPAR downregulation primarily at low P(r) synapses (Sanderson et al., 2018), it could be that synaptic scaling-down and mGluR-LTD are two manifestations of the similar underlying mechanisms? Indeed, there are other points of similarity between these forms of plasticity. For example, in some conditions, glutamate release is enhanced in mGluR-LTD (Xu et al., 2013), the AMPAR trafficking in synaptic scaling-down occludes that induced by DHPG (Hu et al., 2010) and some molecular mechanisms are utilized in both forms of plasticity, notably tyrosine dephosphorylation (Moult et al., 2006; Hu et al., 2010). However, it is worth noting that not all molecular mechanisms are necessarily shared. For example, no role for Homer 1a has been found in mGluR-LTD (Hu et al., 2010). Also, other forms of synaptic scaling alter the induction of synaptic plasticity by altering the properties of neurotransmitter release (Soares et al., 2017) demonstrating that links between different forms of plasticity may be complex. In summary, it is perhaps reasonable to conclude that some but not all mechanisms may be shared between mGluR-LTD and synaptic scaling-down.

Of significant interest is whether the mechanisms revealed by making these experimental manipulations are engaged in endogenous physiological processes. One exciting possibility is that Homer 1a-induced synaptic scaling-down is engaged during sleep. Synaptic Homer1a is upregulated during sleep where it orchestrates synaptic downregulation as a result of group I mGluR-induced AMPAR trafficking and dephosphorylation (Diering et al., 2017). These findings are consistent with the synapse homeostasis hypothesis that suggests that information is encoded during waking hours via LTP-induced increases in synapse strength, followed by synapse weakening during sleep (Tononi and Cirelli, 2014). Ultrastructural studies suggest that large synapses are spared when synapses undergo weakening during sleep (de Vivo et al., 2017). As structural features of synapses are correlated with their function, e.g., PSD size is correlated with active zone size, which correlates with the number of docked vesicles and P(r) (Dobrunz and Stevens, 1997; Schikorski and Stevens, 1997; Murthy et al., 2001; Holderith et al., 2012), the identity of the stable synapses that are resistant to weakening during sleep may correspond to high P(r) synapses that exhibit lower levels of mGluR1 due to theta burst activity (Sanderson et al., 2018). If so, this would be consistent with Homer1a-mGluR signaling during sleep selectively downregulating low P(r) synapses that express higher levels of mGluR1. In the context of the sleep–wake cycle, the access Homer1a has to the synapse may be gated by noradrenergic and adenosine signaling (Diering et al., 2017), and so these additional regulatory mechanisms will also determine the extent of mGluR activation and consequent weakening of synapses.

### Heterosynaptic Plasticity

An additional potential mechanism by which mGluRs could be activated in a way that depends on P(r) is an input non-specific way through heterosynaptic signaling. If LTP is induced at a cluster of synapses, as has been observed to occur *in vivo* in response to sensory experience (Makino and Malinow, 2011), neighboring non-conditioned synapses become downregulated due to mGluR activity (Oh et al., 2015; Winnubst et al., 2015). This downregulation involves removal of AMPARs from synapses, suggested by SEP-GluA2 imaging, and also spine shrinkage. The location of the mGluR trigger for the heterosynaptic signaling has not been defined and may be at the conditioned synapses, and a diffusible signaling molecule may diffuse to neighboring non-conditioned synapses. Alternatively, the group I mGluR may be located at the non-conditioned synapse and be activated via non-glutamatergic signaling, for example, the IEG Homer 1a. The second scenario is consistent with low P(r) synapses that express higher levels of group I mGluRs but that are activated less often, being more susceptible to downregulation via heterosynaptic signaling.

Interestingly, using inducible presynaptic expression of tetanus toxin light chain to suppress transmitter release, the IEG Arc was found to be trafficked specifically to synapses with reduced activity, in a process termed inverse synaptic tagging (Okuno et al., 2012). The trafficking occurred via an interaction with CAMKIIβ and the extent of the Arc enrichment correlated with AMPAR removal occurring at those synapses. As Arc is also involved in mGluR-LTD (Waung et al., 2008), it raises the possibility that low activity synapses are specialized for downregulation via mGluR-dependent signaling of the kind that is recruited in mGluR-LTD. To test this hypothesis, the expression levels and activation of other signaling molecules involved in mGluR-LTD (Gladding et al., 2009b; Sanderson et al., 2016) could be investigated with respect to synaptic activity levels.

## Role of Retrograde Messengers in Coordinating the Pre- and Post-Synapse

**FIGURE 3:**
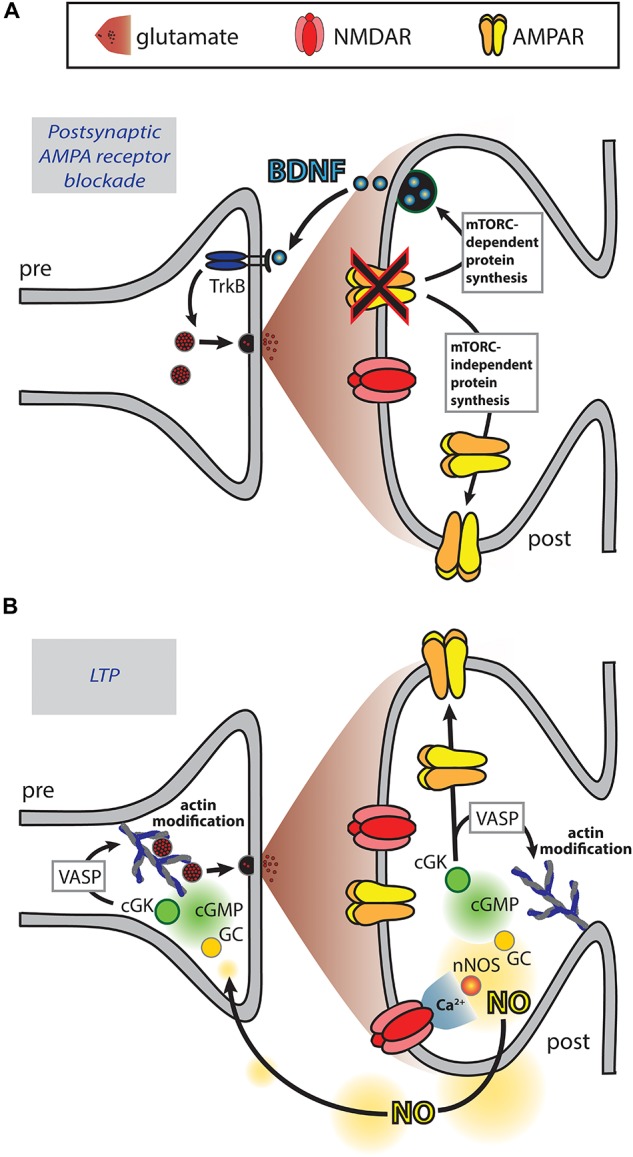
Retrograde messengers may coordinate pre- and post-synaptic function. **(A)** Blockade of postsynaptic AMPARs results in mTORC-dependent protein translation of BDNF that acts as a retrograde messenger to enhance presynaptic release. Additionally, mTORC-independent protein translation results in increased postsynaptic AMPAR expression (Thiagarajan et al., 2005; Jakawich et al., 2010; Lindskog et al., 2010; Henry et al., 2012, 2018). **(B)** During LTP induction, calcium influx through NMDARs activates neuronal nitric oxide synthase (nNOS), which catalyzes the production of nitric oxide (NO). NO diffuses to the pre- and post-synaptic compartments where it activates the guanylate cyclase (GC), cGMP, cGMP-dependent protein kinase (cGK) signaling cascade, resulting in enhanced presynaptic synaptophysin puncta and postsynaptic AMPAR puncta (Antonova et al., 2001; Wang et al., 2005). At both the pre- and post-synapse cGK activates VASP, a protein that plays a role in actin modification. Additionally, cGK phosphorylates the AMPAR subunit GluA1 driving enhanced surface expression (Serulle et al., 2007).

In addition to the mechanisms detailed above, signals that involve retrograde messengers may coordinate the pre- and post-synapse. For example, these may be of relevance to the increase in presynaptic activity induced by overexpression of postsynaptic scaffolding proteins (El-Husseini et al., 2000; Sala et al., 2001). In particular, the role of retrograde messengers in coordinating the pre- and post-synapse has been studied with reference to the increase in synaptic strength that can be induced by pharmacological blockade of post-synapse function, a manipulation that may be of relevance to sensory impairment or neural damage for example as a result of stroke (Thiagarajan et al., 2005). These studies have found that brain derived neurotrophic factor (BDNF) may act as a retrograde messenger, synthesized at the post-synapse in response to phospholipase D and mammalian target of rapamycin complex 1 (mTORC1) signaling (Jakawich et al., 2010; Lindskog et al., 2010; Henry et al., 2012, 2018) (Figure 3A). These presynaptic changes occur simultaneously with increases in postsynaptic AMPAR number via mTORC-independent protein synthesis.

The adaptations in response to postsynaptic receptor blockade typically occur over the time frame of hours (Thiagarajan et al., 2005; Jakawich et al., 2010; Lindskog et al., 2010; Henry et al., 2012). However, molecular alterations that could underlie the functional correlation between the pre- and post-synapse occur much more rapidly. Retrograde messengers were first proposed as a means by which LTP, thought to be triggered by the activation of postsynaptic NMDARs, may result in a persistent increase in neurotransmitter release. NO is thought to act as a retrograde messenger in LTP (Zhuo et al., 1993) and its fast diffusion time may allow activity-dependent increases in function at the pre- and the post-synapse to be coordinated. For example, immunofluorescence imaging has revealed that NMDAR-dependent chemical LTP induces NO signaling that results in increased expression of synaptic markers simultaneously at both the pre- and the post-synapse leading to increased levels of co-localization between them (Antonova et al., 2001; Wang et al., 2005). These effects were found to be mediated via the guanosine 3′,5′ cyclic monophosphate (cGMP)-dependent signaling pathway leading to phosphorylation of the actin regulator VASP. As these and other cGMP-dependent effects are observed at both the pre- and post-synapse (Wang et al., 2005; Sanderson and Sher, 2013), NO-induced cGMP signaling could occur in both compartments to bring about coordinated changes (Figure 3B). Actin-dependent modification of spine structure may undergo bidirectional modulation via cAMP and cGMP signals (see review by Borovac et al., 2018) and so possibly these mechanisms could contribute to modifying spine structure, as well as modifying the expression of synaptic proteins, to ensure that the pre- and post-synapse are matched. Numerous other signaling molecules and potentially even ions such as potassium act as retrograde messengers in LTP (Bliss and Collingridge, 1993; Regehr et al., 2009) and may therefore also coordinate changes in pre- and post-synaptic function.

There is evidence that mGluR-LTD is expressed via both pre- and post-synaptic changes (Fitzjohn et al., 2001; Snyder et al., 2001; Xiao et al., 2001; Rouach and Nicoll, 2003; Tan et al., 2003; Huang et al., 2004; Moult et al., 2006; Gladding et al., 2009a; Casimiro et al., 2011; Sanderson et al., 2011, 2018; Eales et al., 2014), with pre-synaptic changes triggered via activation of post-synaptically expressed mGluRs and release of retrograde messengers such as 12-lipoxygenase metabolites of arachidonic acid (Watabe et al., 2002; Feinmark et al., 2003). In this form of plasticity, it is possible that pre- and post-synaptic changes may be coordinated which may result in the two compartments remaining functionally matched. mGluRs also induce structural changes at spines (Kamikubo et al., 2006; Shinoda et al., 2010; Hasegawa et al., 2015) possibly by regulating the actin cytoskeleton via a mechanism that involves an interaction between GluA2 and N-cadherin leading to the actin regulator cofilin (Zhou et al., 2011). Therefore, mGluR function may also be involved in triggering changes to the structure of synapses as well as their molecular composition, and so may play a role in ensuring these two aspects of synapse physiology are in step.

In addition to diffusible signaling molecules, precise co-ordination between the pre- and post-synapse occurs via direct physical interactions. The matching of AMPARs with the presynaptic release machinery in trans-synaptic molecular “nanocolumns” (Tang et al., 2016) depends on the C-terminal region of Neuroligin-1 and if its function is perturbed, then synaptic transmission is diminished (Haas et al., 2018). This is proposed to be due to Neuroligin-1 performing a linking role between presynaptic neurexins, which it binds via its extracellular N-terminal region, and PSD-95-anchored AMPAR nanodomains, which it binds via its intracellular C-terminal domain.

In summary, there appear to be multiple signaling mechanisms and dedicated molecular machinery that could result in coordination between changes at the pre- and post-synapse.

## Patterning of Synaptic Connections

Above we have summarized data indicating that the pre- and the post-synapse are structurally and functionally correlated, that multiple mechanisms may bring this about including mGluR signaling, and that the expression of mGluRs may play a role in patterning the modulation of synapse strength in several physiological situations. It is not clear how synapses with certain physiological characteristics, including P(r), are arranged on the dendritic trees of excitatory neurons. Are dendrites structured such that synapses with certain properties are located in specific locations? A range of studies have addressed this question and found that synapses diminish in size toward the ends of basal and apical oblique dendrites (Katz et al., 2009; Grillo et al., 2018), while increasing along the somato apical-dendritic axis (Magee and Cook, 2000). The arrangement of synaptic properties may have implications for the integration of synaptic inputs at proximal and distal locations. For example, at proximal locations inputs require strict temporal coincidence in order to sum linearly, whereas at distal locations inputs are amplified more strongly without the need for precise coincidence (Branco and Hausser, 2011). This could lead to proximal and distal dendrites processing different streams of information: Temporally coded information at proximal dendrites and rate based information at distal dendrites (Branco and Hausser, 2011). These synaptic integration properties may be influenced by differential calcium responses at different dendritic locations (Walker et al., 2017).

The properties of neighboring synapses have been examined using similar imaging techniques to those used to investigate intra-synaptic properties. For example, measurement of P(r) using FM dyes has been used to reveal that neighboring synapses on the same dendritic branch have very similar P(r) and that the P(r) is set by the local activity level (Branco et al., 2008). This results in a negative correlation between the density of synaptic contacts and their P(r) and that directly modulating dendritic depolarization can influence P(r), both locally and globally. Neighboring synaptic inputs have been observed to exhibit correlated activity over a range of developmental time points when examined both *in vitro* and *in vivo* (Kleindienst et al., 2011; Takahashi et al., 2012; Winnubst et al., 2015; Wilson et al., 2016; Iacaruso et al., 2017; Scholl et al., 2017). It is therefore plausible that similar P(r) at neighboring synapses may be induced by similar endogenous activity at neighboring co-active synapses.

At the post-synapse, mechanisms also exist that may result in neighboring synapses having similar characteristics. One such mechanism may be calcium-induced calcium release, as in developing synapses this can result in enhanced potentiation at coincidentally active neighboring spines, resulting in clustered synapse maturation (Lee et al., 2016). Conversely, synapses that neighbor a group of co-active synapses, but that are not coincidentally active themselves, are weakened (Oh et al., 2015; Winnubst et al., 2015). Mechanisms such as these may contribute to the clustered postsynaptic enhancement of synapses *in vivo* that occurs in response to sensory experience (Makino and Malinow, 2011).

Astrocytes may also play a role in regulating synaptic P(r), since when astrocytic function is perturbed the P(r) of heterosynaptic inputs become less divergent, implying that astrocytes play a role in maintaining heterogeneity of P(r) over the entire cell (Letellier et al., 2016). The investigation on the role of astrocytes on P(r) utilized two heterosynaptic inputs, which would be unlikely to make synapses that neighbor each other. Therefore presumably the role astrocytes play in maintaining heterogeneity of P(r) is not “local” and is therefore distinct from the mechanisms that ensures similarity of P(r) of neighboring synapses (Branco et al., 2008).

## Future Directions and Prospects

A major area of neuroscience research is aimed at understanding the processes involved in synaptic plasticity, the most extensively studied of which is NMDAR-dependent LTP (Bliss and Collingridge, 1993). Although it is well established that this form of LTP involves both pre- and post-synaptic alterations, including changes in P(r) as well as AMPAR number and properties (Bliss and Collingridge, 2013), these pre- and post-synaptic processes are usually studied in isolation. Our recent finding that P(r) can affect the re-distribution of AMPARs adds an extra layer of complexity to the understanding of plastic events at the level of the single synapse. The induction of LTP by TBS triggers an initial short-term potentiation (STP) component that is mediated by an increase in P(r). It would be predicted that this would result in an internalization of some of the mGluR1 that may be present at the synapse and thereby protect the synapse from postsynaptic weakening mediated by this receptor. This is turn would help stabilize AMPARs that are inserted during LTP. In contrast, in the absence of STP the AMPARs that are inserted during LTP could be more labile since they would be more susceptible to mGluR1-mediated synaptic weakening. At CA3–CA1 principal synapses, mGluR1 is expressed predominantly early in development where it may contribute the refinement of hippocampal synaptic connectivity. Indeed, early in development at these synapses, LTP is predominantly mediated by an increase in P(r) (Palmer et al., 2004), though this changes to a postsynaptically dominated LTP mechanism via a switch triggered by presynaptic kainate receptors (Lauri et al., 2006).

At certain other synapses, such as the parallel synapses between granule cells and Purkinje cells, mGluR1 is the trigger for LTD in adult tissue (Aiba et al., 1994; Conquet et al., 1994) and postsynaptic mechanisms appear to dominate (Wang and Linden, 2000). Whether similar mechanisms to rapidly coordinate pre- and post-synaptic functionality operate at these cerebellar synapses and elsewhere in the CNS remains to be determined.

In summary, rapidly coordinated changes in pre- and post-synaptic activity, mediated by the actions of the neurotransmitter itself, are likely to impact on many facets of synaptic transmission and plasticity in health and disease. These are areas ripe for future investigation.

## Author Contributions

The review was written by TS and edited by JG and GC.

## Conflict of Interest

The authors declare that the research was conducted in the absence of any commercial or financial relationships that could be construed as a potential conflict of interest.

## References

[B1] Abrahamsson T. Chou C. Y. C. Li S. Y. Mancino A. Costa R. P. Brock J. A. (2017). Differential regulation of evoked and spontaneous release by presynaptic NMDA Receptors. *Neuron* 96 839–855 e835. 29033205 10.1016/j.neuron.2017.09.030

[B2] Aiba A. Chen C. Herrup K. Rosenmund C. Stevens C. F. Tonegawa S. (1994). Reduced hippocampal long-term potentiation and context-specific deficit in associative learning in mGluR1 mutant mice. *Cell* 79 365–375. 10.1016/0092-8674(94)90204-6 7954802

[B3] Allen N. J. Eroglu C. (2017). Cell biology of astrocyte-synapse interactions. *Neuron* 96 697–708. 10.1016/j.neuron.2017.09.056 29096081 PMC5687890

[B4] Ango F. Prezeau L. Muller T. Tu J. C. Xiao B. Worley P. F. (2001). Agonist-independent activation of metabotropic glutamate receptors by the intracellular protein Homer. *Nature* 411 962–965. 10.1038/35082096 11418862

[B5] Antonova I. Arancio O. Trillat A. C. Wang H. G. Zablow L. Udo H. (2001). Rapid increase in clusters of presynaptic proteins at onset of long-lasting potentiation. *Science* 294 1547–1550. 10.1126/science.1066273 11641465

[B6] Araki Y. Lin D. T. Huganir R. L. (2010). Plasma membrane insertion of the AMPA receptor GluA2 subunit is regulated by NSF binding and Q/R editing of the ion pore. *Proc. Natl. Acad. Sci. U.S.A.* 107 11080–11085. 10.1073/pnas.1006584107 20534470 PMC2890737

[B7] Araki Y. Zeng M. Zhang M. Huganir R. L. (2015). Rapid dispersion of SynGAP from synaptic spines triggers AMPA receptor insertion and spine enlargement during LTP. *Neuron* 85 173–189. 10.1016/j.neuron.2014.12.023 25569349 PMC4428669

[B8] Araya R. Vogels T. P. Yuste R. (2014). Activity-dependent dendritic spine neck changes are correlated with synaptic strength. *Proc. Natl. Acad. Sci. U.S.A.* 111 E2895–E2904. 10.1073/pnas.1321869111 24982196 PMC4104910

[B9] Ashby M. C. De La Rue S. A. Ralph G. S. Uney J. Collingridge G. L. Henley J. M. (2004). Removal of AMPA receptors (AMPARs) from synapses is preceded by transient endocytosis of extrasynaptic AMPARs. *J. Neurosci.* 24 5172–5176. 10.1523/jneurosci.1042-04.2004 15175386 PMC3309030

[B10] Ashby M. C. Maier S. R. Nishimune A. Henley J. M. (2006). Lateral diffusion drives constitutive exchange of AMPA receptors at dendritic spines and is regulated by spine morphology. *J. Neurosci.* 26 7046–7055. 10.1523/jneurosci.1235-06.2006 16807334 PMC6673929

[B11] Beique J. C. Lin D. T. Kang M. G. Aizawa H. Takamiya K. Huganir R. L. (2006). Synapse-specific regulation of AMPA receptor function by PSD-95. *Proc. Natl. Acad. Sci. U.S.A.* 103 19535–19540. 10.1073/pnas.0608492103 17148601 PMC1748260

[B12] Betz W. J. Bewick G. S. (1992). Optical analysis of synaptic vesicle recycling at the frog neuromuscular junction. *Science* 255 200–203. 10.1126/science.1553547 1553547

[B13] Betz W. J. Mao F. Bewick G. S. (1992). Activity-dependent fluorescent staining and destaining of living vertebrate motor nerve terminals. *J. Neurosci.* 12 363–375. 10.1523/jneurosci.12-02-00363.1992 1371312 PMC6575621

[B14] Bliss T. V. Collingridge G. L. (1993). A synaptic model of memory: long-term potentiation in the hippocampus. *Nature* 361 31–39. 10.1038/361031a0 8421494

[B15] Bliss T. V. Collingridge G. L. (2013). Expression of NMDA receptor-dependent LTP in the hippocampus: bridging the divide. *Mol Brain* 6:5. 10.1186/1756-6606-6-5 23339575 PMC3562207

[B16] Borovac J. Bosch M. Okamoto K. (2018). Regulation of actin dynamics during structural plasticity of dendritic spines: signaling messengers and actin-binding proteins. *Mol. Cell Neurosci.* 91 122–130. 10.1016/j.mcn.2018.07.001 30004015

[B17] Brakeman P. R. Lanahan A. A. O’Brien R. Roche K. Barnes C. A. Huganir R. L. (1997). Homer: a protein that selectively binds metabotropic glutamate receptors. *Nature* 386 284–288. 10.1038/386284a0 9069287

[B18] Branco T. Hausser M. (2011). Synaptic integration gradients in single cortical pyramidal cell dendrites. *Neuron* 69 885–892. 10.1016/j.neuron.2011.02.006 21382549 PMC6420135

[B19] Branco T. Staras K. Darcy K. J. Goda Y. (2008). Local dendritic activity sets release probability at hippocampal synapses. *Neuron* 59 475–485. 10.1016/j.neuron.2008.07.006 18701072 PMC6390949

[B20] Brodin L. Low P. Shupliakov O. (2000). Sequential steps in clathrin-mediated synaptic vesicle endocytosis. *Curr. Opin. Neurobiol.* 10 312–320. 10.1016/s0959-4388(00)00097-0 10851177

[B21] Casimiro T. M. Sossa K. G. Uzunova G. Beattie J. B. Marsden K. C. Carroll R. C. (2011). mGluR and NMDAR activation internalize distinct populations of AMPARs. *Mol. Cell. Neurosci.* 48 161–170. 10.1016/j.mcn.2011.07.007 21807099 PMC3163744

[B22] Charlesworth P. Cotterill E. Morton A. Grant S. G. Eglen S. J. (2015). Quantitative differences in developmental profiles of spontaneous activity in cortical and hippocampal cultures. *Neural Dev.* 10:1. 10.1186/s13064-014-0028-0 25626996 PMC4320829

[B23] Conquet F. Bashir Z. I. Davies C. H. Daniel H. Ferraguti F. Bordi F. (1994). Motor deficit and impairment of synaptic plasticity in mice lacking mGluR1. *Nature* 372 237–243. 10.1038/372237a0 7969468

[B24] Dallerac G. Zapata J. Rouach N. (2018). Versatile control of synaptic circuits by astrocytes: where, when and how? *Nat. Rev. Neurosci.* 19 729–743. 10.1038/s41583-018-0080-6 30401802

[B25] de Vivo L. Bellesi M. Marshall W. Bushong E. A. Ellisman M. H. Tononi G. (2017). Ultrastructural evidence for synaptic scaling across the wake/sleep cycle. *Science* 355 507–510. 10.1126/science.aah5982 28154076 PMC5313037

[B26] Diering G. H. Nirujogi R. S. Roth R. H. Worley P. F. Pandey A. Huganir R. L. (2017). Homer1a drives homeostatic scaling-down of excitatory synapses during sleep. *Science* 355 511–515. 10.1126/science.aai8355 28154077 PMC5382711

[B27] Dobrunz L. E. Stevens C. F. (1997). Heterogeneity of release probability, facilitation, and depletion at central synapses. *Neuron* 18 995–1008. 10.1016/s0896-6273(00)80338-4 9208866

[B28] Eales K. L. Palygin O. O’Loughlin T. Rasooli-Nejad S. Gaestel M. Muller J. (2014). The MK2/3 cascade regulates AMPAR trafficking and cognitive flexibility. *Nat. Commun.* 5:4701. 10.1038/ncomms5701 25134715 PMC4143933

[B29] El-Husseini A. E. Schnell E. Chetkovich D. M. Nicoll R. A. Bredt D. S. (2000). PSD-95 involvement in maturation of excitatory synapses. *Science* 290 1364–1368. 11082065

[B30] Emptage N. Bliss T. V. Fine A. (1999). Single synaptic events evoke NMDA receptor-mediated release of calcium from internal stores in hippocampal dendritic spines. *Neuron* 22 115–124. 10.1016/s0896-6273(00)80683-2 10027294

[B31] Emptage N. J. Reid C. A. Fine A. Bliss T. V. (2003). Optical quantal analysis reveals a presynaptic component of LTP at hippocampal Schaffer-associational synapses. *Neuron* 38 797–804. 10.1016/s0896-6273(03)00325-8 12797963

[B32] Enoki R. Hu Y. L. Hamilton D. Fine A. (2009). Expression of long-term plasticity at individual synapses in hippocampus is graded, bidirectional, and mainly presynaptic: optical quantal analysis. *Neuron* 62 242–253. 10.1016/j.neuron.2009.02.026 19409269

[B33] Feinmark S. J. Begum R. Tsvetkov E. Goussakov I. Funk C. D. Siegelbaum S. A. (2003). 12-lipoxygenase metabolites of arachidonic acid mediate metabotropic glutamate receptor-dependent long-term depression at hippocampal CA3-CA1 synapses. *J. Neurosci.* 23 11427–11435. 10.1523/jneurosci.23-36-11427.2003 14673007 PMC6740529

[B34] Fitzjohn S. M. Palmer M. J. May J. E. Neeson A. Morris S. A. Collingridge G. L. (2001). A characterisation of long-term depression induced by metabotropic glutamate receptor activation in the rat hippocampus in vitro. *J. Physiol.* 537 421–430. 10.1111/j.1469-7793.2001.00421.x 11731575 PMC2278956

[B35] Fukata Y. Dimitrov A. Boncompain G. Vielemeyer O. Perez F. Fukata M. (2013). Local palmitoylation cycles define activity-regulated postsynaptic subdomains. *J. Cell Biol.* 202 145–161. 10.1083/jcb.201302071 23836932 PMC3704990

[B36] Gladding C. M. Collett V. J. Jia Z. Bashir Z. I. Collingridge G. L. Molnar E. (2009a). Tyrosine dephosphorylation regulates AMPAR internalisation in mGluR-LTD. *Mol. Cell. Neurosci.* 40 267–279. 10.1016/j.mcn.2008.10.014 19063969

[B37] Gladding C. M. Fitzjohn S. M. Molnar E. (2009b). Metabotropic glutamate receptor-mediated long-term depression: molecular mechanisms. *Pharmacol. Rev.* 61 395–412. 10.1124/pr.109.001735 19926678 PMC2802426

[B38] Glasgow S. D. McPhedrain R. Madranges J. F. Kennedy T. E. Ruthazer E. S. (2019). Approaches and limitations in the investigation of synaptic transmission and plasticity. *Front. Synaptic Neurosci.* 11:20. 10.3389/fnsyn.2019.00020 31396073 PMC6667546

[B39] Grillo F. W. Neves G. Walker A. Vizcay-Barrena G. Fleck R. A. Branco T. (2018). A distance-dependent distribution of presynaptic boutons tunes frequency-dependent dendritic integration. *Neuron* 99 275–282 e273. 10.1016/j.neuron.2018.06.015 29983327 PMC6078905

[B40] Haas K. T. Compans B. Letellier M. Bartol T. M. Grillo-Bosch D. Sejnowski T. J. (2018). Pre-post synaptic alignment through neuroligin-1 tunes synaptic transmission efficiency. *eLife* 7:e31755 30044218 10.7554/eLife.31755 PMC6070337

[B41] Harata N. Ryan T. A. Smith S. J. Buchanan J. Tsien R. W. (2001). Visualizing recycling synaptic vesicles in hippocampal neurons by FM 1-43 photoconversion. *Proc. Natl. Acad. Sci. U.S.A.* 98 12748–12753. 10.1073/pnas.171442798 11675506 PMC60125

[B42] Harris K. M. Stevens J. K. (1989). Dendritic spines of CA 1 pyramidal cells in the rat hippocampus: serial electron microscopy with reference to their biophysical characteristics. *J. Neurosci.* 9 2982–2997. 10.1523/jneurosci.09-08-02982.1989 2769375 PMC6569708

[B43] Harvey C. D. Yasuda R. Zhong H. Svoboda K. (2008). The spread of Ras activity triggered by activation of a single dendritic spine. *Science* 321 136–140. 10.1126/science.1159675 18556515 PMC2745709

[B44] Hasegawa S. Sakuragi S. Tominaga-Yoshino K. Ogura A. (2015). Dendritic spine dynamics leading to spine elimination after repeated inductions of LTD. *Sci. Rep.* 5:7707. 10.1038/srep07707 25573377 PMC4648349

[B45] Heine M. Groc L. Frischknecht R. Beique J. C. Lounis B. Rumbaugh G. (2008). Surface mobility of postsynaptic AMPARs tunes synaptic transmission. *Science* 320 201–205. 10.1126/science.1152089 18403705 PMC2715948

[B46] Henneberger C. Bard L. Panatier A. Reynolds J. P. Medvedev N. I. Minge D. (2018). LTP induction drives remodeling of astroglia to boost glutamate escape from synapses. *BioRxiv [Preprint]*

[B47] Henry F. E. McCartney A. J. Neely R. Perez A. S. Carruthers C. J. Stuenkel E. L. (2012). Retrograde changes in presynaptic function driven by dendritic mTORC1. *J. Neurosci.* 32 17128–17142. 10.1523/JNEUROSCI.2149-12.2012 23197706 PMC3518308

[B48] Henry F. E. Wang X. Serrano D. Perez A. S. Carruthers C. J. L. Stuenkel E. L. (2018). A Unique Homeostatic Signaling Pathway Links Synaptic Inactivity to Postsynaptic mTORC1. *J. Neurosci.* 38 2207–2225. 10.1523/JNEUROSCI.1843-17.2017 29311141 PMC5830511

[B49] Hessler N. A. Shirke A. M. Malinow R. (1993). The probability of transmitter release at a mammalian central synapse. *Nature* 366 569–572. 10.1038/366569a0 7902955

[B50] Holderith N. Lorincz A. Katona G. Rozsa B. Kulik A. Watanabe M. (2012). Release probability of hippocampal glutamatergic terminals scales with the size of the active zone. *Nat. Neurosci.* 15 988–997. 10.1038/nn.3137 22683683 PMC3386897

[B51] Hruska M. Henderson N. Le Marchand S. J. Jafri H. Dalva M. B. (2018). Synaptic nanomodules underlie the organization and plasticity of spine synapses. *Nat. Neurosci.* 21 671–682. 10.1038/s41593-018-0138-9 29686261 PMC5920789

[B52] Hu J. H. Park J. M. Park S. Xiao B. Dehoff M. H. Kim S. (2010). Homeostatic scaling requires group I mGluR activation mediated by Homer1a. *Neuron* 68 1128–1142. 10.1016/j.neuron.2010.11.008 21172614 PMC3013614

[B53] Huang B. Wang W. Bates M. Zhuang X. (2008). Three-dimensional super-resolution imaging by stochastic optical reconstruction microscopy. *Science* 319 810–813. 10.1126/science.1153529 18174397 PMC2633023

[B54] Huang C. C. You J. L. Wu M. Y. Hsu K. S. (2004). Rap1-induced p38 mitogen-activated protein kinase activation facilitates AMPA receptor trafficking via the GDI.Rab5 complex. Potential role in (S)-3,5-dihydroxyphenylglycene-induced long term depression. *J. Biol. Chem.* 279 12286–12292. 10.1074/jbc.m312868200 14709549

[B55] Iacaruso M. F. Gasler I. T. Hofer S. B. (2017). Synaptic organization of visual space in primary visual cortex. *Nature* 547 449–452. 10.1038/nature23019 28700575 PMC5533220

[B56] Jakawich S. K. Nasser H. B. Strong M. J. McCartney A. J. Perez A. S. Rakesh N. (2010). Local presynaptic activity gates homeostatic changes in presynaptic function driven by dendritic BDNF synthesis. *Neuron* 68 1143–1158. 10.1016/j.neuron.2010.11.034 21172615 PMC3046391

[B57] Jensen T. P. Zheng K. Cole N. Marvin J. S. Looger L. L. Rusakov D. A. (2019). Multiplex imaging relates quantal glutamate release to presynaptic Ca(2+) homeostasis at multiple synapses in situ. *Nat. Commun.* 10:1414. 10.1038/s41467-019-09216-8 30926781 PMC6441074

[B58] Johnstone V. P. Raymond C. R. (2013). Postsynaptic protein synthesis is required for presynaptic enhancement in persistent forms of long-term potentiation. *Front. Synaptic Neurosci.* 5:1. 10.3389/fnsyn.2013.00001 23450328 PMC3582942

[B59] Kamikubo Y. Egashira Y. Tanaka T. Shinoda Y. Tominaga-Yoshino K. Ogura A. (2006). Long-lasting synaptic loss after repeated induction of LTD: independence to the means of LTD induction. *Eur. J. Neurosci.* 24 1606–1616. 10.1111/j.1460-9568.2006.05032.x 17004924

[B60] Katz Y. Menon V. Nicholson D. A. Geinisman Y. Kath W. L. Spruston N. (2009). Synapse distribution suggests a two-stage model of dendritic integration in CA1 pyramidal neurons. *Neuron* 63 171–177. 10.1016/j.neuron.2009.06.023 19640476 PMC2921807

[B61] Kavalali E. T. (2015). The mechanisms and functions of spontaneous neurotransmitter release. *Nat. Rev. Neurosci.* 16 5–16. 10.1038/nrn3875 25524119

[B62] Kay A. R. Alfonso A. Alford S. Cline H. T. Holgado A. M. Sakmann B. (1999). Imaging synaptic activity in intact brain and slices with FM1-43 in *C. elegans*, lamprey, and rat. *Neuron* 24 809–817. 10.1016/s0896-6273(00)81029-6 10624945

[B63] Kay L. Humphreys L. Eickholt B. J. Burrone J. (2011). Neuronal activity drives matching of pre- and postsynaptic function during synapse maturation. *Nat. Neurosci.* 14 688–690. 10.1038/nn.2826 21532580

[B64] Kim J. Goldsberry M. E. Harmon T. C. Freeman J. H. (2016). Developmental changes in hippocampal ca1 single neuron firing and theta activity during associative learning. *PLoS One* 11:e0164781. 10.1371/journal.pone.0164781 27764172 PMC5072650

[B65] Kleindienst T. Winnubst J. Roth-Alpermann C. Bonhoeffer T. Lohmann C. (2011). Activity-dependent clustering of functional synaptic inputs on developing hippocampal dendrites. *Neuron* 72 1012–1024. 10.1016/j.neuron.2011.10.015 22196336

[B66] Kopec C. D. Li B. Wei W. Boehm J. Malinow R. (2006). Glutamate receptor exocytosis and spine enlargement during chemically induced long-term potentiation. *J. Neurosci.* 26 2000–2009. 10.1523/jneurosci.3918-05.2006 16481433 PMC6674938

[B67] Kopec C. D. Real E. Kessels H. W. Malinow R. (2007). GluR1 links structural and functional plasticity at excitatory synapses. *J. Neurosci.* 27 13706–13718. 10.1523/jneurosci.3503-07.2007 18077682 PMC6673607

[B68] Lauri S. E. Vesikansa A. Segerstrale M. Collingridge G. L. Isaac J. T. Taira T. (2006). Functional maturation of CA1 synapses involves activity-dependent loss of tonic kainate receptor-mediated inhibition of glutamate release. *Neuron* 50 415–429. 10.1016/j.neuron.2006.03.020 16675396

[B69] Lee K. F. Soares C. Thivierge J. P. Beique J. C. (2016). Correlated synaptic inputs drive dendritic calcium amplification and cooperative plasticity during clustered synapse development. *Neuron* 89 784–799. 10.1016/j.neuron.2016.01.012 26853305

[B70] Letellier M. Park Y. K. Chater T. E. Chipman P. H. Gautam S. G. Oshima-Takago T. (2016). Astrocytes regulate heterogeneity of presynaptic strengths in hippocampal networks. *Proc. Natl. Acad. Sci. U.S.A.* 113 E2685–E2694. 10.1073/pnas.1523717113 27118849 PMC4868440

[B71] Lin D. T. Huganir R. L. (2007). PICK1 and phosphorylation of the glutamate receptor 2 (GluR2) AMPA receptor subunit regulates GluR2 recycling after NMDA receptor-induced internalization. *J. Neurosci.* 27 13903–13908. 10.1523/jneurosci.1750-07.2007 18077702 PMC6673624

[B72] Lin D. T. Makino Y. Sharma K. Hayashi T. Neve R. Takamiya K. (2009). Regulation of AMPA receptor extrasynaptic insertion by 4.1*N, phosphorylation and palmitoylation*. *Nat Neurosci* 12 879–887. 10.1038/nn.2351 19503082 PMC2712131

[B73] Lindskog M. Li L. Groth R. D. Poburko D. Thiagarajan T. C. Han X. (2010). Postsynaptic GluA1 enables acute retrograde enhancement of presynaptic function to coordinate adaptation to synaptic inactivity. *Proc. Natl. Acad. Sci. U.S.A.* 107 21806–21811. 10.1073/pnas.1016399107 21098665 PMC3003060

[B74] MacGillavry H. D. Song Y. Raghavachari S. Blanpied T. A. (2013). Nanoscale scaffolding domains within the postsynaptic density concentrate synaptic AMPA receptors. *Neuron* 78 615–622. 10.1016/j.neuron.2013.03.009 23719161 PMC3668352

[B75] Magee J. C. Cook E. P. (2000). Somatic EPSP amplitude is independent of synapse location in hippocampal pyramidal neurons. *Nat. Neurosci.* 3 895–903. 10.1038/78800 10966620

[B76] Makino H. Malinow R. (2011). Compartmentalized versus global synaptic plasticity on dendrites controlled by experience. *Neuron* 72 1001–1011. 10.1016/j.neuron.2011.09.036 22196335 PMC3310180

[B77] Matsuzaki M. Honkura N. Ellis-Davies G. C. Kasai H. (2004). Structural basis of long-term potentiation in single dendritic spines. *Nature* 429 761–766. 10.1038/nature02617 15190253 PMC4158816

[B78] Miesenbock G. De Angelis D. A. Rothman J. E. (1998). Visualizing secretion and synaptic transmission with pH-sensitive green fluorescent proteins. *Nature* 394 192–195. 10.1038/28190 9671304

[B79] Mitchell D. E. Martineau E. Tazerart S. Araya R. (2019). Probing single synapses via the photolytic release of neurotransmitters. *Front. Synaptic Neurosci.* 11:19. 10.3389/fnsyn.2019.00019 31354469 PMC6640007

[B80] Moult P. R. Gladding C. M. Sanderson T. M. Fitzjohn S. M. Bashir Z. I. Molnar E. (2006). Tyrosine phosphatases regulate AMPA receptor trafficking during metabotropic glutamate receptor-mediated long-term depression. *J. Neurosci.* 26 2544–2554. 10.1523/jneurosci.4322-05.2006 16510732 PMC6793648

[B81] Murthy V. N. Schikorski T. Stevens C. F. Zhu Y. (2001). Inactivity produces increases in neurotransmitter release and synapse size. *Neuron* 32 673–682. 10.1016/s0896-6273(01)00500-1 11719207

[B82] Murthy V. N. Sejnowski T. J. Stevens C. F. (1997). Heterogeneous release properties of visualized individual hippocampal synapses. *Neuron* 18 599–612. 10.1016/s0896-6273(00)80301-3 9136769

[B83] Nadif Kasri N. Nakano-Kobayashi A. Van Aelst L. (2011). Rapid synthesis of the X-linked mental retardation protein OPHN1 mediates mGluR-dependent LTD through interaction with the endocytic machinery. *Neuron* 72 300–315. 10.1016/j.neuron.2011.09.001 22017989 PMC3206629

[B84] Nair D. Hosy E. Petersen J. D. Constals A. Giannone G. Choquet D. (2013). Super-resolution imaging reveals that AMPA receptors inside synapses are dynamically organized in nanodomains regulated by PSD95. *J. Neurosci.* 33 13204–13224. 10.1523/JNEUROSCI.2381-12.2013 23926273 PMC6619720

[B85] Noguchi J. Matsuzaki M. Ellis-Davies G. C. Kasai H. (2005). Spine-neck geometry determines NMDA receptor-dependent Ca2+ signaling in dendrites. *Neuron* 46 609–622. 10.1016/j.neuron.2005.03.015 15944129 PMC4151245

[B86] Oh W. C. Parajuli L. K. Zito K. (2015). Heterosynaptic structural plasticity on local dendritic segments of hippocampal CA1 neurons. *Cell Rep.* 10 162–169. 10.1016/j.celrep.2014.12.016 25558061 PMC4294981

[B87] Okuno H. Akashi K. Ishii Y. Yagishita-Kyo N. Suzuki K. Nonaka M. (2012). Inverse synaptic tagging of inactive synapses via dynamic interaction of Arc/Arg3.1 with CaMKIIbeta. *Cell* 149 886–898. 10.1016/j.cell.2012.02.062 22579289 PMC4856149

[B88] Oliet S. H. Malenka R. C. Nicoll R. A. (1997). Two distinct forms of long-term depression coexist in CA1 hippocampal pyramidal cells. *Neuron* 18 969–982. 10.1016/s0896-6273(00)80336-0 9208864

[B89] Padamsey Z. Tong R. Emptage N. (2017). Glutamate is required for depression but not potentiation of long-term presynaptic function. *eLife* 6:e29688. 10.7554/eLife.29688 29140248 PMC5714480

[B90] Padamsey Z. Tong R. Emptage N. (2019). Optical quantal analysis using Ca(2+) indicators: a robust method for assessing transmitter release probability at excitatory synapses by imaging single glutamate release events. *Front. Synaptic Neurosci.* 11:5. 10.3389/fnsyn.2019.00005 30886576 PMC6409341

[B91] Palmer M. J. Irving A. J. Seabrook G. R. Jane D. E. Collingridge G. L. (1997). The group I mGlu receptor agonist DHPG induces a novel form of LTD in the CA1 region of the hippocampus. *Neuropharmacology* 36 1517–1532. 10.1016/s0028-3908(97)00181-0 9517422

[B92] Palmer M. J. Isaac J. T. Collingridge G. L. (2004). Multiple, developmentally regulated expression mechanisms of long-term potentiation at CA1 synapses. *J. Neurosci.* 24 4903–4911. 10.1523/jneurosci.0170-04.2004 15163681 PMC6729367

[B93] Passafaro M. Nakagawa T. Sala C. Sheng M. (2003). Induction of dendritic spines by an extracellular domain of AMPA receptor subunit GluR2. *Nature* 424 677–681. 10.1038/nature01781 12904794

[B94] Patterson M. A. Szatmari E. M. Yasuda R. (2010). AMPA receptors are exocytosed in stimulated spines and adjacent dendrites in a Ras-ERK-dependent manner during long-term potentiation. *Proc. Natl. Acad. Sci. U.S.A.* 107 15951–15956. 10.1073/pnas.0913875107 20733080 PMC2936631

[B95] Pyle J. L. Kavalali E. T. Choi S. Tsien R. W. (1999). Visualization of synaptic activity in hippocampal slices with FM1-43 enabled by fluorescence quenching. *Neuron* 24 803–808. 10.1016/s0896-6273(00)81028-4 10624944

[B96] Regehr W. G. Carey M. R. Best A. R. (2009). Activity-dependent regulation of synapses by retrograde messengers. *Neuron* 63 154–170. 10.1016/j.neuron.2009.06.021 19640475 PMC3251517

[B97] Rollenhagen A. Lubke J. H. (2006). The morphology of excitatory central synapses: from structure to function. *Cell Tissue Res.* 326 221–237. 10.1007/s00441-006-0288-z 16932936

[B98] Rose C. R. Felix L. Zeug A. Dietrich D. Reiner A. Henneberger C. (2017). Astroglial glutamate signaling and uptake in the hippocampus. *Front. Mol. Neurosci.* 10:451. 10.3389/fnmol.2017.00451 29386994 PMC5776105

[B99] Rosenmund C. Clements J. D. Westbrook G. L. (1993). Nonuniform probability of glutamate release at a hippocampal synapse. *Science* 262 754–757. 10.1126/science.7901909 7901909

[B100] Rosenmund C. Stevens C. F. (1996). Definition of the readily releasable pool of vesicles at hippocampal synapses. *Neuron* 16 1197–1207. 10.1016/s0896-6273(00)80146-4 8663996

[B101] Rouach N. Nicoll R. A. (2003). Endocannabinoids contribute to short-term but not long-term mGluR-induced depression in the hippocampus. *Eur. J. Neurosci.* 18 1017–1020. 10.1046/j.1460-9568.2003.02823.x 12925027

[B102] Ryan T. A. Reuter H. Wendland B. Schweizer F. E. Tsien R. W. Smith S. J. (1993). The kinetics of synaptic vesicle recycling measured at single presynaptic boutons. *Neuron* 11 713–724. 10.1016/0896-6273(93)90081-2 8398156

[B103] Sala C. Piech V. Wilson N. R. Passafaro M. Liu G. Sheng M. (2001). Regulation of dendritic spine morphology and synaptic function by Shank and Homer. *Neuron* 31 115–130. 10.1016/s0896-6273(01)00339-7 11498055

[B104] Sanderson T. M. Bradley C. A. Georgiou J. Hong Y. H. Ng A. N. Lee Y. (2018). The probability of neurotransmitter release governs AMPA receptor trafficking via activity-dependent regulation of mGluR1 surface expression. *Cell Rep* 25 3631–3646 e3633. 10.1016/j.celrep.2018.12.010 30590038 PMC6315206

[B105] Sanderson T. M. Collingridge G. L. Fitzjohn S. M. (2011). Differential trafficking of AMPA receptors following activation of NMDA receptors and mGluRs. *Mol. Brain* 4:30. 10.1186/1756-6606-4-30 21794146 PMC3160366

[B106] Sanderson T. M. Hogg E. L. Collingridge G. L. Correa S. A. (2016). Hippocampal metabotropic glutamate receptor long-term depression in health and disease: focus on mitogen-activated protein kinase pathways. *J. Neurochem.* 139(Suppl. 2), 200–214. 10.1111/jnc.13592 26923875

[B107] Sanderson T. M. Sher E. (2013). The role of phosphodiesterases in hippocampal synaptic plasticity. *Neuropharmacology* 74 86–95. 10.1016/j.neuropharm.2013.01.011 23357335

[B108] Sankaranarayanan S. De Angelis D. Rothman J. E. Ryan T. A. (2000). The use of pHluorins for optical measurements of presynaptic activity. *Biophys. J.* 79 2199–2208. 10.1016/s0006-3495(00)76468-x 11023924 PMC1301110

[B109] Schikorski T. Stevens C. F. (1997). Quantitative ultrastructural analysis of hippocampal excitatory synapses. *J. Neurosci.* 17 5858–5867. 10.1523/jneurosci.17-15-05858.1997 9221783 PMC6573206

[B110] Schikorski T. Stevens C. F. (1999). Quantitative fine-structural analysis of olfactory cortical synapses. *Proc. Natl. Acad. Sci. U.S.A.* 96 4107–4112. 10.1073/pnas.96.7.4107 10097171 PMC22428

[B111] Scholl B. Wilson D. E. Fitzpatrick D. (2017). Local order within global disorder: synaptic architecture of visual space. *Neuron* 96:e1124. 10.1016/j.neuron.2017.10.017 29103806 PMC5868972

[B112] Serulle Y. Zhang S. Ninan I. Puzzo D. McCarthy M. Khatri L. (2007). A GluR1-cGKII interaction regulates AMPA receptor trafficking. *Neuron* 56 670–688. 10.1016/j.neuron.2007.09.016 18031684 PMC2153457

[B113] Shinoda Y. Tanaka T. Tominaga-Yoshino K. Ogura A. (2010). Persistent synapse loss induced by repetitive LTD in developing rat hippocampal neurons. *PLoS One* 5:e10390. 10.1371/journal.pone.0010390 20436928 PMC2861005

[B114] Snyder E. M. Philpot B. D. Huber K. M. Dong X. Fallon J. R. Bear M. F. (2001). Internalization of ionotropic glutamate receptors in response to mGluR activation. *Nat. Neurosci.* 4 1079–1085. 10.1038/nn746 11687813

[B115] Soares C. Lee K. F. H. Beique J. C. (2017). Metaplasticity at CA1 synapses by homeostatic control of presynaptic release dynamics. *Cell Rep.* 21 1293–1303. 10.1016/j.celrep.2017.10.025 29091767

[B116] Soares C. Trotter D. Longtin A. Beique J. C. Naud R. (2019). Parsing out the variability of transmission at central synapses using optical quantal analysis. *Front. Synaptic Neurosci.* 11:22. 10.3389/fnsyn.2019.00022 31474847 PMC6702664

[B117] Takahashi N. Kitamura K. Matsuo N. Mayford M. Kano M. Matsuki N. (2012). Locally synchronized synaptic inputs. *Science* 335 353–356. 10.1126/science.1210362 22267814

[B118] Takumi Y. Ramirez-Leon V. Laake P. Rinvik E. Ottersen O. P. (1999). Different modes of expression of AMPA and NMDA receptors in hippocampal synapses. *Nat. Neurosci.* 2 618–624. 10.1038/10172 10409387

[B119] Tan Y. Hori N. Carpenter D. O. (2003). The mechanism of presynaptic long-term depression mediated by group I metabotropic glutamate receptors. *Cell Mol. Neurobiol.* 23 187–203. 12735631 10.1023/A:1022949922364 PMC11530151

[B120] Tanaka J. Horiike Y. Matsuzaki M. Miyazaki T. Ellis-Davies G. C. Kasai H. (2008). Protein synthesis and neurotrophin-dependent structural plasticity of single dendritic spines. *Science* 319 1683–1687. 10.1126/science.1152864 18309046 PMC4218863

[B121] Tanaka J. Matsuzaki M. Tarusawa E. Momiyama A. Molnar E. Kasai H. (2005). Number and density of AMPA receptors in single synapses in immature cerebellum. *J. Neurosci.* 25 799–807. 10.1523/jneurosci.4256-04.2005 15673659 PMC6725634

[B122] Tang A. H. Chen H. Li T. P. Metzbower S. R. MacGillavry H. D. Blanpied T. A. (2016). A trans-synaptic nanocolumn aligns neurotransmitter release to receptors. *Nature* 536 210–214. 10.1038/nature19058 27462810 PMC5002394

[B123] Thiagarajan T. C. Lindskog M. Tsien R. W. (2005). Adaptation to synaptic inactivity in hippocampal neurons. *Neuron* 47 725–737. 10.1016/j.neuron.2005.06.037 16129401

[B124] Thorsen T. S. Madsen K. L. Rebola N. Rathje M. Anggono V. Bach A. (2010). Identification of a small-molecule inhibitor of the PICK1 PDZ domain that inhibits hippocampal LTP and LTD. *Proc. Natl. Acad. Sci. U.S.A.* 107 413–418. 10.1073/pnas.0902225107 20018661 PMC2806717

[B125] Tokuoka H. Goda Y. (2008). Activity-dependent coordination of presynaptic release probability and postsynaptic GluR2 abundance at single synapses. *Proc. Natl. Acad. Sci. U.S.A.* 105 14656–14661. 10.1073/pnas.0805705105 18794522 PMC2567165

[B126] Tononi G. Cirelli C. (2014). Sleep and the price of plasticity: from synaptic and cellular homeostasis to memory consolidation and integration. *Neuron* 81 12–34. 10.1016/j.neuron.2013.12.025 24411729 PMC3921176

[B127] Tracy T. E. Yan J. J. Chen L. (2011). Acute knockdown of AMPA receptors reveals a trans-synaptic signal for presynaptic maturation. *EMBO J.* 30 1577–1592. 10.1038/emboj.2011.59 21378752 PMC3102285

[B128] Turrigiano G. G. (2008). The self-tuning neuron: synaptic scaling of excitatory synapses. *Cell* 135 422–435. 10.1016/j.cell.2008.10.008 18984155 PMC2834419

[B129] Voglmaier S. M. Kam K. Yang H. Fortin D. L. Hua Z. Nicoll R. A. (2006). Distinct endocytic pathways control the rate and extent of synaptic vesicle protein recycling. *Neuron* 51 71–84. 10.1016/j.neuron.2006.05.027 16815333

[B130] Walker A. S. Neves G. Grillo F. Jackson R. E. Rigby M. O’Donnell C. (2017). Distance-dependent gradient in NMDAR-driven spine calcium signals along tapering dendrites. *Proc. Natl. Acad. Sci. U.S.A.* 114 E1986–E1995. 10.1073/pnas.1607462114 28209776 PMC5347575

[B131] Walmsley B. Alvarez F. J. Fyffe R. E. (1998). Diversity of structure and function at mammalian central synapses. *Trends Neurosci.* 21 81–88. 10.1016/s0166-2236(97)01170-3 9498304

[B132] Wang H. G. Lu F. M. Jin I. Udo H. Kandel E. R. de Vente J. (2005). Presynaptic and postsynaptic roles of NO, cGK, and RhoA in long-lasting potentiation and aggregation of synaptic proteins. *Neuron* 45 389–403. 10.1016/j.neuron.2005.01.011 15694326

[B133] Wang Y. T. Linden D. J. (2000). Expression of cerebellar long-term depression requires postsynaptic clathrin-mediated endocytosis. *Neuron* 25 635–647. 10.1016/s0896-6273(00)81066-1 10774731

[B134] Ward B. McGuinness L. Akerman C. J. Fine A. Bliss T. V. Emptage N. J. (2006). State-dependent mechanisms of LTP expression revealed by optical quantal analysis. *Neuron* 52 649–661. 10.1016/j.neuron.2006.10.007 17114049

[B135] Watabe A. M. Carlisle H. J. O’Dell T. J. (2002). Postsynaptic induction and presynaptic expression of group 1 mGluR-dependent LTD in the hippocampal CA1 region. *J. Neurophysiol.* 87 1395–1403. 10.1152/jn.00723.2001 11877514

[B136] Watanabe S. Rost B. R. Camacho-Perez M. Davis M. W. Sohl-Kielczynski B. Rosenmund C. (2013). Ultrafast endocytosis at mouse hippocampal synapses. *Nature* 504 242–247. 10.1038/nature12809 24305055 PMC3957339

[B137] Waung M. W. Pfeiffer B. E. Nosyreva E. D. Ronesi J. A. Huber K. M. (2008). Rapid translation of Arc/Arg3.1 selectively mediates mGluR-dependent LTD through persistent increases in AMPAR endocytosis rate. *Neuron* 59 84–97. 10.1016/j.neuron.2008.05.014 18614031 PMC2580055

[B138] Willig K. I. Kellner R. R. Medda R. Hein B. Jakobs S. Hell S. W. (2006). Nanoscale resolution in GFP-based microscopy. *Nat. Methods* 3 721–723. 10.1038/nmeth922 16896340

[B139] Wilson D. E. Whitney D. E. Scholl B. Fitzpatrick D. (2016). Orientation selectivity and the functional clustering of synaptic inputs in primary visual cortex. *Nat. Neurosci.* 19 1003–1009. 10.1038/nn.4323 27294510 PMC5240628

[B140] Winnubst J. Cheyne J. E. Niculescu D. Lohmann C. (2015). Spontaneous activity drives local synaptic plasticity in vivo. *Neuron* 87 399–410. 10.1016/j.neuron.2015.06.029 26182421

[B141] Xiao M. Y. Zhou Q. Nicoll R. A. (2001). Metabotropic glutamate receptor activation causes a rapid redistribution of AMPA receptors. *Neuropharmacology* 41 664–671. 10.1016/s0028-3908(01)00134-4 11640920

[B142] Xu W. Tse Y. C. Dobie F. A. Baudry M. Craig A. M. Wong T. P. (2013). Simultaneous monitoring of presynaptic transmitter release and postsynaptic receptor trafficking reveals an enhancement of presynaptic activity in metabotropic glutamate receptor-mediated long-term depression. *J. Neurosci.* 33 5867–5877. 10.1523/JNEUROSCI.1508-12.2013 23536098 PMC4138313

[B143] Yudowski G. A. Puthenveedu M. A. Leonoudakis D. Panicker S. Thorn K. S. Beattie E. C. (2007). Real-time imaging of discrete exocytic events mediating surface delivery of AMPA receptors. *J. Neurosci.* 27 11112–11121. 10.1523/jneurosci.2465-07.2007 17928453 PMC3249441

[B144] Zhang J. Wang Y. Chi Z. Keuss M. J. Pai Y. M. Kang H. C. (2011). The AAA+ ATPase Thorase regulates AMPA receptor-dependent synaptic plasticity and behavior. *Cell* 145 284–299. 10.1016/j.cell.2011.03.016 21496646 PMC3085003

[B145] Zhang Y. Cudmore R. H. Lin D. T. Linden D. J. Huganir R. L. (2015). Visualization of NMDA receptor-dependent AMPA receptor synaptic plasticity in vivo. *Nat. Neurosci.* 18 402–407. 10.1038/nn.3936 25643295 PMC4339371

[B146] Zhou Z. Hu J. Passafaro M. Xie W. Jia Z. (2011). GluA2 (GluR2) regulates metabotropic glutamate receptor-dependent long-term depression through N-cadherin-dependent and cofilin-mediated actin reorganization. *J. Neurosci.* 31 819–833. 10.1523/JNEUROSCI.3869-10.2011 21248105 PMC6632944

[B147] Zhuo M. Small S. A. Kandel E. R. Hawkins R. D. (1993). Nitric oxide and carbon monoxide produce activity-dependent long-term synaptic enhancement in hippocampus. *Science* 260 1946–1950. 10.1126/science.8100368 8100368

